# Supplementary material for: A Likelihood Approach for Real-Time Calibration of Stochastic Compartmental Epidemic Models
Source: PLoS Comput Biol. 2017 Jan 17;13(1):e1005257. doi: 10.1371/journal.pcbi.1005257 (PMC5240920; doi:10.1371/journal.pcbi.1005257)
Supplement: S1 File — (TAR.GZ) [file pcbi.1005257.s014.tar.gz › HSPH_Online-SI-Revision/IPoi09/n10-mild/n10-mild_BENCH2_table0.pdf]

1	newly 3 weeks spec	176.	448.333	{320., 593.}	157.164	0.40292	newly 3 weeks cumul	530.	1399.51	{974., 1736.}	167.018	0.42285	newly 5 weeks spec	135.	372.784	{216., 539.}	176.572	0.416234	newly 5 weeks cumul	825.	2195.55	{1592., 2734.}	168.509	0.424367	newly 50 weeks cumul	2160.	4142.86	{2567., 5904.}	91.7992	0.260824
2	newly 3 weeks spec	316.	719.018	{424., 963.}	127.684	0.338226	newly 3 weeks cumul	1222.	2311.68	{1736., 2794.}	91.0071	0.2757	newly 5 weeks spec	183.	527.946	{241., 866.}	188.495	0.415086	newly 5 weeks cumul	1613.	3469.75	{2346., 4282.}	115.112	0.320472	newly 55 weeks cumul	2172.	5112.48	{2948., 7276.}	135.381	0.349289
3	newly 3 weeks spec	434.	371.415	{178., 620.}	41.9804	0.217155	newly 3 weeks cumul	1373.	1676.54	{1028., 2663.}	39.4667	0.146398	newly 5 weeks spec	309.	145.463	{51., 244.}	60.281	0.450649	newly 5 weeks cumul	2076.	2062.07	{1192., 3266.}	35.0216	0.158104	newly 58 weeks cumul	2924.	2271.17	{1250., 3543.}	37.8032	0.209585
4	newly 3 weeks spec	417.	677.456	{430., 848.}	67.2977	0.227448	newly 3 weeks cumul	1378.	2175.04	{1638., 2557.}	63.4189	0.227922	newly 5 weeks spec	196.	507.754	{267., 739.}	160.419	0.39103	newly 5 weeks cumul	1896.	3277.54	{2197., 3920.}	76.4361	0.248582	newly 53 weeks cumul	2527.	5387.61	{3265., 7328.}	113.756	0.313721
5	newly 3 weeks spec	111.	221.044	{122., 322.}	106.266	0.310094	newly 3 weeks cumul	344.	688.333	{415., 935.}	107.978	0.321196	newly 5 weeks spec	94.	194.444	{105., 298.}	108.936	0.295686	newly 5 weeks cumul	547.	1087.87	{603., 1537.}	104.948	0.305412	newly 33 weeks cumul	2021.	2999.04	{1491., 4502.}	57.9603	0.183928
6	newly 3 weeks spec	331.	196.185	{61., 439.}	59.3488	0.384221	newly 3 weeks cumul	1004.	965.556	{387., 1942.}	41.8253	0.196966	newly 5 weeks spec	169.	70.6667	{17., 150.}	70.1512	0.588714	newly 5 weeks cumul	1390.	1156.07	{449., 2300.}	45.473	0.240864	newly 59 weeks cumul	2050.	1249.11	{463., 2426.}	52.4264	0.339123
7	newly 3 weeks spec	277.	529.667	{258., 890.}	95.9807	0.258371	newly 3 weeks cumul	873.	1958.78	{1148., 2811.}	125.997	0.332052	newly 5 weeks spec	186.	288.511	{105., 543.}	76.0454	0.233692	newly 5 weeks cumul	1277.	2647.29	{1463., 4115.}	109.296	0.293114	newly 55 weeks cumul	1892.	3284.09	{1642., 5185.}	79.7862	0.229559
8	newly 3 weeks spec	269.	423.244	{263., 629.}	63.387	0.203652	newly 3 weeks cumul	743.	1433.64	{981., 1958.}	95.3282	0.280318	newly 5 weeks spec	224.	291.556	{154., 488.}	46.1706	0.163135	newly 5 weeks cumul	1216.	2080.04	{1374., 2983.}	75.0658	0.232666	newly 50 weeks cumul	3026.	3218.49	{1856., 5060.}	33.3701	0.142864
9	newly 3 weeks spec	476.	721.197	{494., 870.}	58.3448	0.228511	newly 3 weeks cumul	1520.	2230.31	{1820., 2578.}	54.9234	0.231565	newly 5 weeks spec	288.	568.197	{297., 828.}	102.949	0.301381	newly 5 weeks cumul	2165.	3458.36	{2530., 4069.}	65.8259	0.245284	newly 54 weeks cumul	2749.	5846.41	{3909., 7665.}	112.674	0.314758
10	newly 3 weeks spec	159.	213.722	{91., 474.}	64.7799	0.212182	newly 3 weeks cumul	644.	827.778	{358., 1654.}	53.4765	0.184417	newly 5 weeks spec	135.	123.944	{36., 267.}	67.6955	0.338597	newly 5 weeks cumul	960.	1116.39	{440., 2349.}	54.4271	0.208189	newly 51 weeks cumul	1852.	1443.25	{562., 2953.}	55.644	0.306643
11	newly 3 weeks spec	170.	245.107	{82., 570.}	74.3067	0.249038	newly 3 weeks cumul	592.	1132.11	{461., 2176.}	100.452	0.267652	newly 5 weeks spec	147.	100.179	{27., 234.}	54.1059	0.364365	newly 5 weeks cumul	872.	1393.	{545., 2916.}	78.1291	0.236096	newly 56 weeks cumul	1526.	1545.57	{592., 3248.}	45.5018	0.211209
12	newly 3 weeks spec	240.	976.23	{747., 1190.}	306.762	0.601677	newly 3 weeks cumul	1103.	3178.7	{2642., 3619.}	188.187	0.450392	newly 5 weeks spec	100.	663.557	{427., 1068.}	563.557	0.795873	newly 5 weeks cumul	1384.	4662.89	{3774., 5324.}	236.914	0.523272	newly 58 weeks cumul	1560.	6524.16	{4923., 8516.}	318.216	0.612611
13	newly 3 weeks spec	357.	602.909	{413., 719.}	75.7576	0.277925	newly 3 weeks cumul	1370.	1860.55	{1488., 2161.}	43.0524	0.19694	newly 5 weeks spec	224.	497.818	{327., 720.}	128.555	0.371838	newly 5 weeks cumul	1865.	2916.73	{2180., 3410.}	63.2601	0.246967	newly 50 weeks cumul	2415.	5644.31	{3863., 7430.}	133.719	0.355289
14	newly 3 weeks spec	307.	544.127	{322., 732.}	80.2699	0.248278	newly 3 weeks cumul	1011.	1789.41	{1173., 2245.}	81.0262	0.259221	newly 5 weeks spec	225.	418.952	{207., 657.}	91.0688	0.260148	newly 5 weeks cumul	1501.	2689.87	{1791., 3386.}	82.0141	0.254357	newly 51 weeks cumul	2614.	4595.44	{2820., 6655.}	78.8143	0.234038
15	newly 3 weeks spec	226.	338.825	{170., 516.}	60.1659	0.193968	newly 3 weeks cumul	696.	1258.7	{675., 1833.}	82.8089	0.243752	newly 5 weeks spec	226.	201.225	{84., 341.}	40.0553	0.201268	newly 5 weeks cumul	1140.	1721.98	{872., 2538.}	58.5022	0.18848	newly 53 weeks cumul	3240.	2304.78	{1084., 3553.}	37.314	0.218938
16	newly 3 weeks spec	319.	696.131	{449., 873.}	118.223	0.325291	newly 3 weeks cumul	1150.	2227.	{1549., 2633.}	94.6386	0.280459	newly 5 weeks spec	224.	517.393	{261., 732.}	132.223	0.338088	newly 5 weeks cumul	1670.	3357.85	{2283., 4101.}	101.069	0.2933	newly 54 weeks cumul	2574.	5223.15	{3004., 6863.}	103.151	0.289238
17	newly 3 weeks spec	105.	274.891	{42., 490.}	185.031	0.469557	newly 3 weeks cumul	312.	726.304	{106., 1316.}	160.298	0.448266	newly 5 weeks spec	82.	326.674	{78., 503.}	305.912	0.577248	newly 5 weeks cumul	477.	1352.17	{266., 2302.}	202.744	0.480406	newly 51 weeks cumul	3040.	6531.48	{5397., 7442.}	114.851	0.328624
18	newly 3 weeks spec	181.	314.66	{151., 541.}	78.8997	0.224361	newly 3 weeks cumul	580.	1110.66	{645., 1695.}	92.909	0.263582	newly 5 weeks spec	194.	209.234	{83., 398.}	50.6909	0.212577	newly 5 weeks cumul	959.	1578.38	{821., 2627.}	69.9732	0.209153	newly 51 weeks cumul	1686.	2264.32	{1066., 4000.}	52.6249	0.175945
19	newly 3 weeks spec	266.	426.08	{188., 743.}	72.3008	0.214451	newly 3 weeks cumul	957.	1656.68	{847., 2690.}	77.906	0.230136	newly 5 weeks spec	162.	235.34	{88., 484.}	70.8765	0.225	newly 5 weeks cumul	1331.	2214.92	{1078., 3872.}	74.0195	0.219222	newly 54 weeks cumul	1835.	2759.48	{1289., 5118.}	63.7319	0.197693
20	newly 3 weeks spec	319.	327.854	{160., 652.}	48.207	0.208215	newly 3 weeks cumul	1048.	1354.93	{772., 2083.}	45.1452	0.156773	newly 5 weeks spec	244.	165.293	{46., 265.}	61.4254	0.382897	newly 5 weeks cumul	1558.	1751.78	{940., 3038.}	43.6222	0.172575	newly 55 weeks cumul	2688.	2069.71	{1055., 3592.}	44.1075	0.245382
21	newly 3 weeks spec	299.	697.712	{416., 954.}	133.496	0.348124	newly 3 weeks cumul	1149.	2292.24	{1706., 2809.}	101.233	0.296433	newly 5 weeks spec	209.	497.373	{241., 830.}	139.778	0.339194	newly 5 weeks cumul	1607.	3388.85	{2311., 4217.}	111.226	0.31201	newly 55 weeks cumul	2136.	4905.05	{2876., 7276.}	130.348	0.340164
22	newly 3 weeks spec	242.	561.447	{392., 705.}	132.003	0.353032	newly 3 weeks cumul	748.	1814.15	{1321., 2170.}	142.533	0.37696	newly 5 weeks spec	169.	415.596	{250., 635.}	146.267	0.364634	newly 5 weeks cumul	1116.	2722.89	{1988., 3294.}	143.987	0.377879	newly 51 weeks cumul	2163.	4383.66	{2742., 6171.}	103.402	0.290019
23	newly 3 weeks spec	259.	603.935	{312., 905.}	135.731	0.348397	newly 3 weeks cumul	963.	2181.96	{1347., 2961.}	130.516	0.357944	newly 5 weeks spec	207.	349.913	{155., 555.}	79.101	0.228823	newly 5 weeks cumul	1381.	2998.13	{1712., 4214.}	119.296	0.325678	newly 56 weeks cumul	1616.	3991.24	{2244., 5673.}	146.983	0.36447
24	newly 3 weeks spec	173.	205.667	{91., 474.}	56.4868	0.206287	newly 3 weeks cumul	546.	800.5	{358., 1578.}	60.989	0.185619	newly 5 weeks spec	105.	120.306	{36., 267.}	73.9418	0.292132	newly 5 weeks cumul	774.	1080.56	{440., 2349.}	62.0155	0.194529	newly 52 weeks cumul	1235.	1398.44	{562., 2953.}	59.919	0.228274
25	newly 3 weeks spec	260.	900.862	{444., 1187.}	250.828	0.531908	newly 3 weeks cumul	915.	2582.98	{799., 3361.}	192.234	0.464746	newly 5 weeks spec	241.	753.185	{417., 1083.}	213.201	0.469223	newly 5 weeks cumul	1474.	4183.46	{2385., 5098.}	187.278	0.449989	newly 59 weeks cumul	2031.	6903.37	{4691., 8606.}	239.9	0.522309
26	newly 3 weeks spec	226.	295.342	{145., 497.}	46.9842	0.163379	newly 3 weeks cumul	712.	1104.03	{662., 1688.}	61.2323	0.192219	newly 5 weeks spec	102.	184.658	{97., 398.}	86.6099	0.224223	newly 5 weeks cumul	971.	1522.16	{849., 2484.}	63.5427	0.195163	newly 50 weeks cumul	1344.	2098.92	{1091., 4187.}	65.9716	0.193758
27	newly 3 weeks spec	316.	645.717	{448., 818.}	113.107	0.341086	newly 3 weeks cumul	1146.	1964.43	{1627., 2377.}	83.6155	0.297761	newly 5 weeks spec	192.	548.817	{314., 784.}	187.283	0.43519	newly 5 weeks cumul	1600.	3116.03	{2290., 3769.}	102.275	0.315563	newly 52 weeks cumul	1907.	6040.42	{3932., 7869.}	216.75	0.484907
28	newly 3 weeks spec	99.	168.297	{61., 302.}	87.3601	0.261451	newly 3 weeks cumul	324.	545.108	{217., 881.}	81.6567	0.248437	newly 5 weeks spec	91.	135.811	{41., 270.}	77.6953	0.25883	newly 5 weeks cumul	522.	833.784	{309., 1422.}	77.115	0.242461	newly 30 weeks cumul	1626.	1857.03	{587., 3727.}	56.052	0.238198
29	newly 3 weeks spec	301.	548.556	{325., 839.}	82.5274	0.234876	newly 3 weeks cumul	953.	1901.37	{1304., 2551.}	99.5142	0.286558	newly 5 weeks spec	234.	360.241	{168., 646.}	69.5552	0.205563	newly 5 weeks cumul	1506.	2710.5	{1704., 3834.}	79.9801	0.235542	newly 53 weeks cumul	2280.	3795.63	{2179., 5952.}	69.0578	0.200068
30	newly 3 weeks spec	342.	282.255	{116., 600.}	48.2145	0.256485	newly 3 weeks cumul	1048.	1114.51	{550., 2203.}	43.8647	0.185507	newly 5 weeks spec	206.	155.745	{55., 324.}	54.4722	0.310997	newly 5 weeks cumul	1528.	1485.32	{697., 2981.}	43.8816	0.202002	newly 52 weeks cumul	2105.	1885.72	{801., 3755.}	46.9884	0.232735
31	newly 3 weeks spec	270.	673.933	{297., 1119.}	152.979	0.362121	newly 3 weeks cumul	910.	2396.71	{1475., 3048.}	163.375	0.403271	newly 5 weeks spec	207.	343.978	{98., 740.}	94.0633	0.280848	newly 5 weeks cumul	1305.	3244.58	{1777., 4636.}	149.1	0.370405	newly 59 weeks cumul	1766.	3817.	{1895., 5969.}	118.982	0.312005
32	newly 3 weeks spec	121.	140.842	{56., 247.}	49.6738	0.200796	newly 3 weeks cumul	377.	477.632	{211., 879.}	51.2495	0.189661	newly 5 weeks spec	116.	108.237	{43., 208.}	45.0318	0.221983	newly 5 weeks cumul	611.	706.789	{305., 1331.}	48.316	0.194701	newly 32 weeks cumul	2466.	1376.03	{547., 2600.}	50.7737	0.352379
33	newly 3 weeks spec	364.	700.6	{490., 844.}	99.0201	0.317532	newly 3 weeks cumul	1353.	2075.6	{1065., 2469.}	64.6859	0.249117	newly 5 weeks spec	266.	608.333	{369., 801.}	131.83	0.366113	newly 5 weeks cumul	1933.	3346.67	{2402., 3926.}	79.2309	0.271457	newly 53 weeks cumul	2442.	6488.93	{4385., 8343.}	165.722	0.413699
34	newly 3 weeks spec	262.	440.048	{260., 664.}	72.7372	0.223086	newly 3 weeks cumul	877.	1590.52	{1067., 2125.}	83.2003	0.250774	newly 5 weeks spec	247.	270.024	{134., 423.}	41.8836	0.171591	newly 5 weeks cumul	1378.	2207.83	{1383., 3196.}	64.3842	0.203939	newly 53 weeks cumul	2645.	2958.6	{1650., 4296.}	33.4405	0.133851
35	newly 3 weeks spec	285.	468.714	{206., 737.}	75.511	0.230277	newly 3 weeks cumul	970.	1711.84	{878., 2491.}	81.4891	0.242639	newly 5 weeks spec	184.	289.127	{107., 442.}	76.0956	0.23635	newly 5 weeks cumul	1379.	2375.54	{1133., 3480.}	79.7104	0.238747	newly 53 weeks cumul	2032.	3225.68	{1443., 4775.}	71.0817	0.219976
36	newly 3 weeks spec	365.	577.122	{292., 956.}	65.2726	0.201898	newly 3 weeks cumul	1132.	2092.69	{1350., 2832.}	85.5196	0.254166	newly 5 weeks spec	206.	340.633	{141., 650.}	78.4724	0.220929	newly 5 weeks cumul	1604.	2879.92	{1689., 4353.}	81.1034	0.238617	newly 55 weeks cumul	2018.	3703.57	{1965., 6227.}	86.0875	0.242527
37	newly 3 weeks spec	233.	506.05	{204., 943.}	123.906	0.302638	newly 3 weeks cumul	842.	1981.98	{975., 3064.}	136.695	0.343553	newly 5 weeks spec	124.	233.025	{63., 533.}	113.286	0.289558	newly 5 weeks cumul	1099.	2570.1	{1191., 4212.}	136.165	0.333788	newly 58 weeks cumul	1455.	2955.55	{1280., 5378.}	108.715	0.279761
38	newly 3 weeks spec	329.	361.534	{161., 816.}	46.1639	0.187132	newly 3 weeks cumul	996.	1384.47	{707., 2452.}	52.4183	0.17432	newly 5 weeks spec	222.	199.103	{80., 457.}	54.4268	0.262577	newly 5 weeks cumul	1520.	1856.78	{897., 3726.}	46.1059	0.168786	newly 54 weeks cumul	2847.	2335.59	{1064., 4854.}	43.4044	0.228118
39	newly 3 weeks spec	207.	709.673	{424., 954.}	242.837	0.517597	newly 3 weeks cumul	1062.	2320.75	{1793., 2793.}	118.526	0.33325	newly 5 weeks spec	83.	497.827	{235., 850.}	499.791	0.731434	newly 5 weeks cumul	1296.	3431.54	{2360., 4280.}	164.779	0.411744	newly 55 weeks cumul	1395.	4862.52	{2948., 7097.}	248.568	0.520641
40	newly 3 weeks spec	80.	177.032	{101., 250.}	124.194	0.326343	newly 3 weeks cumul	250.	558.548	{356., 732.}	125.226	0.332298	newly 5 weeks spec	81.	150.194	{82., 217.}	91.3182	0.257814	newly 5 weeks cumul	408.	873.452	{524., 1167.}	116.248	0.312989	newly 28 weeks cumul	1434.	2172.48	{1000., 3989.}	69.7553	0.215342
41	newly 3 weeks spec	318.	992.	{601., 1306.}	214.454	0.486563	newly 3 weeks cumul	1244.	3189.67	{2431., 3826.}	159.493	0.415944	newly 5 weeks spec	73.	638.07	{295., 1092.}	774.069	0.89183	newly 5 weeks cumul	1452.	4651.58	{3253., 5642.}	221.923	0.500072	newly 59 weeks cumul	1481.	6166.67	{3760., 8210.}	316.385	0.605159
42	newly 3 weeks spec	264.	266.105	{113., 462.}	37.9785	0.174205	newly 3 weeks cumul	803.	1027.87	{499., 1568.}	44.4812	0.161721	newly 5 weeks spec	207.	155.5	{58., 305.}	46.6692	0.275204	newly 5 weeks cumul	1233.	1386.55	{649., 2240.}	39.034	0.15921	newly 51 weeks cumul	1934.	1824.82	{795., 3146.}	38.3838	0.184592
43	newly 3 weeks spec	267.	879.565	{593., 1134.}	229.425	0.505458	newly 3 weeks cumul	1014.	2896.65	{2330., 3369.}	185.666	0.452011	newly 5 weeks spec	120.	588.029	{319., 993.}	390.024	0.657019	newly 5 weeks cumul	1409.	4226.55	{3153., 5064.}	199.968	0.470837	newly 57 weeks cumul	1476.	5814.22	{3933., 7935.}	293.917	0.582308
44	newly 3 weeks spec	266.	542.956	{347., 774.}	105.171	0.29596	newly 3 weeks cumul	845.	1853.56	{1376., 2285.}	119.403	0.331283	newly 5 weeks spec	232.	355.556	{175., 592.}	61.4368	0.189672	newly 5 weeks cumul	1322.	2657.07	{1810., 3361.}	101.846	0.293786	newly 53 weeks cumul	2625.	3689.22	{2219., 5407.}	47.1856	0.159476
45	newly 3 weeks spec	490.	1154.05	{803., 1448.}	136.738	0.365229	newly 3 weeks cumul	1749.	3649.31	{3136., 4102.}	111.966	0.328816	newly 5 weeks spec	208.	717.828	{400., 1066.}	245.11	0.50811	newly 5 weeks cumul	2301.	5303.88	{4326., 6047.}	130.769	0.358101	newly 60 weeks cumul	2545.	7019.2	{5179., 8601.}	175.804	0.433114
46	newly 3 weeks spec	320.	544.609	{252., 888.}	78.2745	0.231035	newly 3 weeks cumul	1046.	2090.17	{1269., 2914.}	100.266	0.280538	newly 5 weeks spec	217.	278.348	{103., 500.}	64.977	0.235253	newly 5 weeks cumul	1558.	2770.67	{1549., 4108.}	80.146	0.233917	newly 57 weeks cumul	1999.	3323.15	{1715., 5133.}	72.2024	0.21366
47	newly 3 weeks spec	449.	870.353	{554., 1038.}	101.533	0.310133	newly 3 weeks cumul	1325.	2396.46	{1092., 3030.}	92.5583	0.299931	newly 5 weeks spec	304.	807.353	{553., 1031.}	165.577	0.412623	newly 5 weeks cumul	2024.	4056.74	{2788., 4650.}	105.518	0.312671	newly 57 weeks cumul	2952.	7717.18	{6069., 9051.}	161.422	0.412841
48	newly 3 weeks spec	220.	750.097	{576., 885.}	240.953	0.520237	newly 3 weeks cumul	864.	2271.26	{1845., 2617.}	165.748	0.418412	newly 5 weeks spec	200.	605.968	{402., 826.}	202.984	0.464746	newly 5 weeks cumul	1267.	3567.58	{2881., 4104.}	181.577	0.441044	newly 54 weeks cumul	1825.	6082.19	{4469., 7619.}	233.271	0.512448
49	newly 3 weeks spec	192.	261.207	{95., 529.}	64.6372	0.22202	newly 3 weeks cumul	594.	954.207	{395., 1716.}	73.0059	0.221906	newly 5 weeks spec	164.	174.138	{56., 349.}	62.4474	0.271233	newly 5 weeks cumul	949.	1341.24	{527., 2611.}	61.7238	0.203938	newly 50 weeks cumul	2640.	1860.76	{655., 3614.}	48.0251	0.295881
50	newly 3 weeks spec	145.	201.816	{78., 367.}	61.6515	0.205181	newly 3 weeks cumul	506.	805.132	{333., 1313.}	72.1188	0.221791	newly 5 weeks spec	93.	116.842	{36., 242.}	62.2524	0.235717	newly 5 weeks cumul	715.	1077.47	{423., 1860.}	67.6187	0.214977	newly 50 weeks cumul	1140.	1397.82	{545., 2524.}	51.9414	0.193769
